# Activation of GABA_B_ receptors in central amygdala attenuates activity of PKCδ + neurons and suppresses punishment-resistant alcohol self-administration in rats

**DOI:** 10.1038/s41386-023-01543-1

**Published:** 2023-02-04

**Authors:** Esi Domi, Li Xu, Sanne Toivainen, Joost Wiskerke, Andrea Coppola, Lovisa Holm, Eric Augier, Michele Petrella, Markus Heilig

**Affiliations:** 1 Center for Social and Affective Neuroscience, Department of Biomedical and Clinical Sciences, Linköping University, S-581 85, Linköping, Sweden; 2 Present Address: School of Pharmacy, Pharmacology Unit, Center for Neuroscience, University of Camerino, Camerino, 62032 Italy

**Keywords:** Addiction, Neuroscience

## Abstract

Alcohol use despite negative consequences is a core phenomenon of alcohol addiction. We recently used alcohol self-administration that is resistant to footshock punishment as a model of this behavior, and found that activity of PKCδ + GABAergic neurons in the central amygdala (CeA) is a determinant of individual susceptibility for punishment resistance. In the present study, we examined whether activation of GABA_B_ receptors in CeA can attenuate the activity of PKCδ + neurons in this region, and whether this will result in suppression of punishment- resistant alcohol self-administration in the minority of rats that show this behavior. Systemic administration of the clinically approved GABA_B_ agonist baclofen (1 and 3 mg/kg) dose- dependently reduced punishment-resistant alcohol self-administration. Bilateral microinjections of baclofen into CeA (64 ng in 0.3 µl/side) reduced the activity of PKCδ + neurons, as measured by Fos expression. This manipulation also selectively suppressed punished alcohol self-administration in punishment-resistant rats. Expression analysis indicated that virtually all CeA PKCδ + neurons express the GABA_B_ receptor. Using in vitro electrophysiology, we found that baclofen induced hyperpolarization of CeA neurons, reducing their firing rate in response to depolarizing current injections. Together, our findings provide a potential mechanism that contributes to the clinical efficacy of baclofen in alcohol addiction. Therapeutic use of baclofen itself is limited by problems of tolerance and need for dose escalation. Our findings support a mechanistic rationale for developing novel, improved alcohol addiction medications that target GABA_B_ receptors, and that lack these limitations, such as e.g., GABA_B_ positive allosteric modulators (PAM:s).

## Introduction

Continued substance use despite negative consequences, commonly referred to as “compulsive use”, is a core phenomenon of addictive disorders [1–3]. Similar to other addictions, only a minority of alcohol users transition from controlled to compulsive use [4]. Accordingly, animal models have begun to incorporate individual differences in vulnerability for behaviors thought to model compulsive use [5–9]. For instance, we recently found that a subset of outbred Wistar rats continued to self-administer alcohol despite a contingent electric foot shock, and could thus be operationally classified as “compulsive”. Chemogenetic inhibition of activated PKCδ + neurons within central amygdala (CeA) of these rats markedly suppressed their compulsive self-administration [7]. These findings converged with our prior observations, in which we found punishment resistance in a subset of rats that chose alcohol over a non-drug reward. The latter behavior was also driven by a CeA-mediated mechanism, i.e. low expression of the GABA-transporter GAT-3 [10]. Using another model of compulsive alcohol taking, continued drinking that is insensitive to quinine adulteration and to alternative saccharin reward, reduced GAT-3 expression was then reported in compulsive Sprague–Dawley rats [8].

These findings converge with prior reports of altered GABAergic function in CeA following development of alcohol dependence [11]. Based on these and other observations, it has been suggested that pharmacological interventions with an ability to restore GABA homeostasis in the CeA may offer opportunities for therapeutics with an ability to attenuate compulsive alcohol use in vulnerable individuals [8, 12]. A mechanism with potential to achieve this would be activation of GABA_B_ receptor agonists [12]. In support of this notion, systemic administration of the orthosteric GABA_B_ agonist baclofen, a medication that is clinically approved for the treatment of spasticity, has been shown to reduce alcohol taking that is resistant to quinine adulteration and alternative saccharin reward in rats with GAT‐3 mRNA downregulation in the CeA [8].

Multiple studies, most of which have used baclofen, have previously reported that GABA_B_ receptor activation is also able to attenuate other alcohol addiction-related behaviors in animal models [13]. For instance, they have shown that baclofen attenuates alcohol consumption and motivation for alcohol in non-dependent [14, 15], alcohol-dependent [16], as well as genetically selected alcohol preferring rats [17, 18]. In addition, baclofen has been shown to prevent the acquisition of alcohol drinking behavior [19], and to suppress responding for alcohol associated cues under extinction conditions in Sardinian alcohol-preferring rats (sP), [20]. These reports parallel and are consistent with meta-analytic support for efficacy of baclofen as a clinical alcohol addiction treatment [21]. Despite its efficacy, however, baclofen has major limitations as a therapeutic, i.e. because its chronic dosing frequently results in tolerance, a need for dose escalation, and accompanying adverse events [22]. Baclofen is nevertheless useful as a tool compound to investigate GABA_B_-mediated effects on alcohol-related behaviors, as a basis for developing improved therapeutics targeting this mechanism, such as positive allosteric modulators (PAM:s) [23, 24].

Based on our previous findings that activity of the CeA accounted for the vast majority, or ~75% of variance in compulsive alcohol self-administration [7], in the present study we therefore set out to determine whether GABA_B_ receptor activation using baclofen modulates activity of PKCδ+ CeA neurons associated with punishment-resistant alcohol self-administration. We then examined whether attenuation of this activity following GABA_B_ agonism reduces compulsive self-administration in rats.

## Materials and methods

### Drugs

For systemic injections, racemic baclofen (Sigma-Aldrich) was dissolved in saline and administered intraperitoneally (i.p.) at 0, 1, or 3 mg/kg, in a volume of 1 ml/kg. For intra-CeA injections, baclofen was dissolved in artificial cerebrospinal fluid (aCSF, 3525; TOCRIS) at 1 mM and was administered bilaterally in 0.3 µl, resulting in a dose of 64 ng / CeA. Alcohol solutions were prepared volume/volume (v/v) in tap water from 99% alcohol. Drugs were prepared fresh on the experimental day, and rats were habituated to the route of the administration before the test. For electrophysiology, baclofen was dissolved in milliQ water, and CGP55845 (1248, TOCRIS) was dissolved in 100% dimethyl sulfoxide (DMSO), and they were stocked at −20 °C.

### Animals

Male Wistar rats (Charles River), weighing 250–300 g (7–9 weeks) at the beginning of the experiments, were pair-housed in a controlled environment (21 ^o^C, humidity-controlled, reverse 12 h light-dark cycle). Rats were given free access to food pellets and tap water for the duration of the experiment and were weighed at least once a week. All behavioral testing was conducted during the dark phase of the light-dark cycle. Rats were habituated three times to the respective administration route before each experiment. All procedures were conducted in accordance with the European Union Directive 2010/63/Eu, and the protocol was approved by the Ethics Committee for Animal Care and Use at Linköping University.

### Alcohol self-administration

Operant training and testing were performed in thirty-two identical operant chambers (Med Associates Inc., St Albans, VT, USA; 30.5 × 29.2 × 24.1 cm) housed in sound-attenuating cubicles. Each operant chamber was equipped with two retractable levers positioned laterally to a liquid cup receptacle. A total of 126 drug-naive rats were trained to self-administer 20% (v/v) alcohol without sucrose/saccharin fading as described previously [25–28]. Rats were trained initially to lever press on a fixed ratio 1 (FR1) 5 s time-out (TO) schedule to self-administer 20% alcohol during 30 min sessions. Pressing once on the lever associated with alcohol (active) was reinforced by the delivery of a volume of 100 μl of 20% alcohol in water in the adjacent drinking well and initiated a concomitant 5 s time-out period signaled by the illumination of the cue-light above the lever. Responses on the inactive lever and during the time-out period were recorded but had no programmed consequences. Once a stable self-administration baseline was reached, the sessions were conducted under a fixed ratio FR2 until a stable baseline of lever pressing was achieved (defined as a minimum of 15 sessions and no change greater than 15% in the total number of reinforcers earned during the last 3 sessions). Animals that did not acquire the self-administration procedure or earned less than 10 reinforcers in the baseline of the training sessions were excluded from the study [29].

### Footshock punished alcohol self-administration

Compulsivity was assessed as responding for alcohol when its delivery was associated with a footshock punishment as previously described [7]. Briefly, conditions were identical to baseline self-administration (i.e 30 min sessions), but each completed FR2 ratio (i.e., 2 responses) was paired with a footshock (0.2 mA, 0.5 s), contingent with the delivery of a volume of 100 µl 20% alcohol in water in the adjacent drinking well. To classify rats as punishment-resistant or sensitive, we calculated a resistance score for each rat, calculated as: (punished alcohol deliveries) / (punished alcohol deliveries + mean alcohol deliveries of last 3 non-punished sessions) [6, 7]. The threshold 0.45 was used to classify shock-sensitive and -resistant rats based on a conservative limit of the shock-resistance distribution peak, identified with Hartigans’ test of unimodality (alpha = 0.001), which corresponds to about 20% decrease from baseline, unpunished alcohol self-administration [7]. Rats with a resistance score > 0.45 were classified as “punishment-resistant” while those with a score < 0.45 were classified as “punishment-sensitive”.

### Systemic baclofen injections

Baclofen (0, 1, 3 mg/kg) was administered i.p. 30 min before the beginning of the self-administration session in shock-sensitive and shock-resistant rats (*n* = 14/group), using a within subject Latin-square design. Punished-alcohol self-administration baseline was re-established between drug tests in 3 consecutive sessions.

### Cannula implantation and intracerebral microinjections

Surgeries were performed after 14 days of punished alcohol self-administration. Animals were anesthetized with isoflurane (2–3%, Baxter), and injected with buprenorphine (0.03 mg/kg, s.c.) 30 min before surgery to relieve pain. Ketoprofen (5 mg/kg, s.c.) was injected after surgery and the following day to relieve pain and reduce inflammation. We implanted guide cannulas (26 gauge, Plastics One) 2 mm above the CeA bilaterally using the following coordinates: AP, −2.5 mm; ML, ± 4.5 mm; DV, −6.4 mm. Cannulas were anchored to the skull with jeweler´s screws and dental cement (Paladur, Agnthos, Lidingö, Sweden). Animals were allowed to recover from surgery for one week and were then allowed to re-establish their baseline of punished self-administration.

Rats received baclofen in 0.3 µl/side through 33-gauge injectors (2 mm below the cannula placement) at the rate of 0.15 ul/min, 15 min prior to the punishment session. At the end of the experiments, animals were transcardially perfused, and brains were removed for performing fluorescent immunohistochemistry and verifying the cannula placement.

To verify the injection site and cannula placement, we selected 4 series of sections from each rat, and coronal sections of 40 μm were mounted on slides and stained with Cresyl violet. Placements of the injector were determined through a Leica DMi8 microscope with a 10x objective lens and brightfield images were mapped onto coronal sections of a rat brain stereotaxic atlas [30].

### Saccharin self-administration

Saccharin self-administration was performed as previously described [27, 28], under conditions similar to those of alcohol self-administration. Briefly, a total of 36 drug-naive rats were trained to self-administer 0.2% saccharin in 30 minutes sessions under a FR2 5 s time-out schedule of reinforcement. A total of 15 FR2 sessions were performed to reach a stable baseline (no change greater than 15% in the total number of reinforcers earned during the last 3 sessions). The effect of systemic (0, 1, 3 mg/kg) and central (64 ng/0.3 ul) baclofen injections were tested 30 or 15 min before the respective saccharin self-administration session.

### Locomotor activity

Baseline locomotor activity was measured in sound-attenuated behavioral chambers equipped with an open field (43 × 43 cm) containing infrared beam detectors (Med Associates, St. Albans, VT) as previously described [27]. Horizontal and vertical movements were examined for 30 min (to match the duration of the alcohol self-administration session) under non-habituated conditions, at ambient light level of 190–210 lux. To assess whether the effects of systemic and central baclofen on alcohol were related to motor impairment, locomotor activity was measured in two separate groups of rats (*n* = 27; *n* = 20, respectively) that were pretreated with vehicle or baclofen 30 (systemic) or 15 min (central injections) before the respective locomotor activity session for evaluating the effects of systemic and central baclofen, respectively. To assess whether the interaction of alcohol and baclofen resulted in sedative effects beyond those of baclofen alone, we analyzed the distance traveled (cm) in the operant chambers during the self‐administration sessions, measured using infrared beams built into the operant chambers and spaced 18.1 cm apart. Duplicate events were detected and removed from the total count as previously described [28].

### RNAscope in situ hybridization

A group of 6 drug-naive rats were deeply anesthetized with isoflurane and transcardially perfused with 0.9% saline followed by 4% paraformaldehyde (PFA). Brains were removed and postfixed in 4% PFA 4 h, then transferred into 30% sucrose solution till sinking. Coronal brain sections (14 µm) were obtained using a Leica cryostat and stored in cryoprotectant (20% glycerol and 30% ethylene glycol in 0.1 M PBS).

Multiplex fluorescent in situ hybridization assay was performed using RNAscope® Multiplex Fluorescent Detection Kit v2 (catalog no. 323110, Advanced Cell Diagnostics) according to the user manual for fixed tissue. Briefly, brain sections were washed in PBS, and mounted on slides. They were post-fixed in 10% neutral-buffered formalin (Thermo Fisher Scientific), dehydrated in 50, 70, 100, and 100% ethanol, and treated with RNAscope Hydrogen Peroxide 10 min at room temperature. Sections were incubated with RNAscope Protease III at 40 °C for 30 min in the HybEZ oven (Advanced Cell Diagnostics), followed by incubation with probes for *Prkcd* mRNA (catalog no. 441791, GeneBank accession number NM_011103.3, Advanced Cell Diagnostics) and *Gabbr1* mRNA (catalog no. 546461-C2, GeneBank accession number NM_031028.3, Advanced Cell Diagnostics) at 40 °C for 2 h. Sections were then incubated with Hybridize AMP1, 2 and 3, after which they were developed for HRP-C1 signal with TSA Plus Cyanine 3 (1:3000 diluted, catalog no. NEL744001KT, PerkinElmer) and HRP-C2 signal with TSA Plus Cyanine 5 (1:3000 diluted, catalog no. NEL745001KT, PerkinElmer). After incubation with DAPI for 30 s at room temperature, slides were cover-slipped with the Antifade Mountant (catalog no. P36961, Invitrogen).

Images of *Prkcd* and *Gabbr1* expression were acquired through a Zeiss LSM 800 upright confocal microscope using a 20x objective lens and cells were identified by DAPI staining. For quantification of *Prkcd*, and *Gabbr1* positive cells (*N* = 6 rats) we counted the total cells of the fluorescent signal (fluorescent “dots”) from two hemispheres of 2 sections. A single fluorescent dot represents the signal that was amplified from the specific probe of the target mRNA and positive cells expressing *Prkcd* and/or *Gabbr1* were detected above the intensity threshold of the assay, 15163 and 13621 respectively, using ImageJ software [31, 32]. Estimated cell counts were averaged across sections for quantification of *Prkcd* and *Gabbr1* expression levels. Positive cells were counted for the respective probe or their combination using the to determine the overlap in PKCδ and GABA_B_ receptor expression.

### Fluorescent immunohistochemistry

Rats were anesthetized using isoflurane 90 min after the start of footshock sessions on day 15, and transcardially perfused with 0.9% saline followed by 4% PFA. Brains were removed and postfixed in 4% PFA for 2 h, then transferred into 30% sucrose solution at 4 °C until sinking. Coronal brain sections (20 µm) of the amygdala (AP bregma level of −1.92 to −2.92 mm) were collected using a Leica cryostat and stored in cryoprotectant at −20 °C until further processing.

For Fos and PKCδ immunofluorescent staining, floating brain sections were washed in PBS 3 × 10 min, then blocked in a solution of 4% bovine serum albumin (BSA) and 0.2% Triton X-100 dissolved in PBS for 1 h at room temperature. For labeling Fos and PKCδ the following antibodies were used: rabbit anti-Fos (1:1000, Abcam, ab190289, RRID: AB_2737414), mouse anti-PKCδ (1:500, BD Bioscience, 610398, RRID: AB_397781). Sections were incubated with primary antibodies overnight at 4 °C. After rinsing in PBS three times, the sections were incubated 2 h at room temperature with following secondary antibodies: donkey anti-rabbit Alexa Fluor 488 (1:200, Thermo Fisher Scientific, A-21206, RRID: AB_2535792), goat anti-mouse Alexa Fluor 568 (1:200, Thermo Fisher Scientific, A-11004, RRID: AB_2534072). Sections were rinsed in PBS three times, mounted on slides, and cover-slipped with Antifade Mountant DAPI (Invitrogen, P36962) or the Antifade Mountant (Invitrogen, P36961).

All Fos and PKCδ immunofluorescence images were acquired through a Zeiss LSM 800 upright confocal microscope using a 20x objective lens. We quantified the total number of Fos and PKCδ positive cells in CeA in a manner blinded to the experimental condition. For each rat, labelled cells were quantified from two hemispheres of 3 sections, and we averaged the counts to give a mean number of each immunoreactive cell type. All images were adjusted to match contrast and brightness in Fiji software; cells were identified by DAPI staining.

### Slice preparation and ex vivo electrophysiology

For electrophysiological recordings, behaviorally naïve 8-10 weeks old male Wistar rats (Charles River, Germany) were deeply anesthetized with isoflurane and decapitated. Brains were quickly removed and placed into an ice-cold N-methyl-D-glucamine (NMDG)-based cutting solution [33] containing (in mM): 92 NMDG, 20 HEPES, 25 glucose, 30 NaHCO3, 1.2 NaH2PO4, 2.5 KCl, 5 sodium ascorbate, 3 sodium pyruvate, 2 thiourea, 10 MgSO4, and 0.5 CaCl2 (310 mOsm, pH 7.4 adjusted using HCl). Acute coronal brain slices (250 μm thick) containing the CeA were obtained using a vibratome (Leica VT1200 S, Leica Biosystems Inc., IL, USA). After cutting, slices were transferred to a holding chamber filled with a pre-warmed (∼34 °C) artificial cerebrospinal fluid (aCSF, in mM): 125 NaCl, 2.5 KCl, 1.25 NaH2PO4, 1 MgCl2, 11 glucose, 26 NaHCO3, 2.4 CaCl2 (310 mOsm, pH 7.4). Subsequently, the solution was maintained at room temperature, and after > 1 h of recovery, a single slice was transferred to the recording chamber and continuously perfused with warmed (∼30–32 °C) aCSF solution at a flow rate of ∼2.0 ml/min. All solutions were saturated with 95% O_2_ and 5% CO_2_. Neurons were visualized with infrared differential interference contrast (IR-DIC) optics on a Zeiss Examiner A1 microscope (Carl Zeiss AB, Stockholm, Sweden) and recordings were aimed at centrolateral amygdala (CeL) neurons. Electrophysiological recordings were carried out using borosilicate glass patch pipettes (2.5–3.0 MΩ; Harvard Apparatus, MA, USA) containing (in mM): 135 K-gluconate, 20 KCl, 10 HEPES, 0.1 EGTA, 2 MgCl_2_, 2 Mg-ATP, 0.3 Na-GTP (290 mOsm, pH adjusted to 7.3 using KOH). Recordings were performed using a Multiclamp 700B amplifier (Molecular Devices, CA, USA), digitalized with a Digidata 1440 A (Molecular Devices, CA, USA; 2 kHz low-pass Bessel filter and acquired 20 kHz), and acquired and analyzed with pClamp 10.7 software (Molecular Devices, CA, USA). The estimated junction potential was 11 mV and was not compensated during electrophysiological recordings.

### Statistical analysis

All data were analyzed with STATISTICA, Stat Soft 13.0 (RRID:SCR_014213), using Student’s *t*-test or analysis of variance (ANOVA), with factors and degrees of freedom for the respective analysis indicated in conjunction with its results. Statistically significant difference was set at *P* < 0.05. *Post-hoc* analyses were conducted when appropriate using Newman-Keuls test. The data are presented as the mean ± SEM. Prior to ANOVA, data were examined for significant violations for assumptions of homogeneity of variance and normality of distribution using Levene’s and Shapiro-Wilk test, respectively. Where homogeneity of variance or normality were significantly violated, data were square root transformed.

## Results

### Baclofen suppresses punished alcohol self-administration in shock-resistant rats

After 14 days of footshock punished alcohol self-administration, 26/64 rats (40%) were classified as punishment-resistant, i.e. had a resistance score > 0.45 [7], while remaining rats were sensitive by this criterion. Two-way ANOVA with group as a between-group factor (levels: punishment- resistant, punishment-sensitive) and treatment as a within-subject factor (levels: 0, 1, 3 mg/kg) confirmed that the two groups differed in their self-administration rates under punished conditions (main effect of group - resistance score: F_1,26_ = 5.3, *p* < 0.05; eta^2^ = 0.17; punished reinforcers obtained: F_1,26_ = 13.2, *p* < 0.001; eta^2^ = 0.34). There was a significant main effect of treatment (resistance score: F_2,52_ = 32.2, *p* < 0.001; eta^2^ = 0.55; punished reinforcers obtained: F_2,52_ = 69.6, *p* < 0.001; eta^2^ = 0.73), and the two groups responded differentially to treatment (group x treatment interaction – resistance score: F_2,52_ = 5.72, *p* < 0.01; eta^2^ = 0.18; punished reinforcers obtained: F_2,52_ = 14.8, *p* < 0.001; eta^2^ = 0.36).

**Fig. 1:**
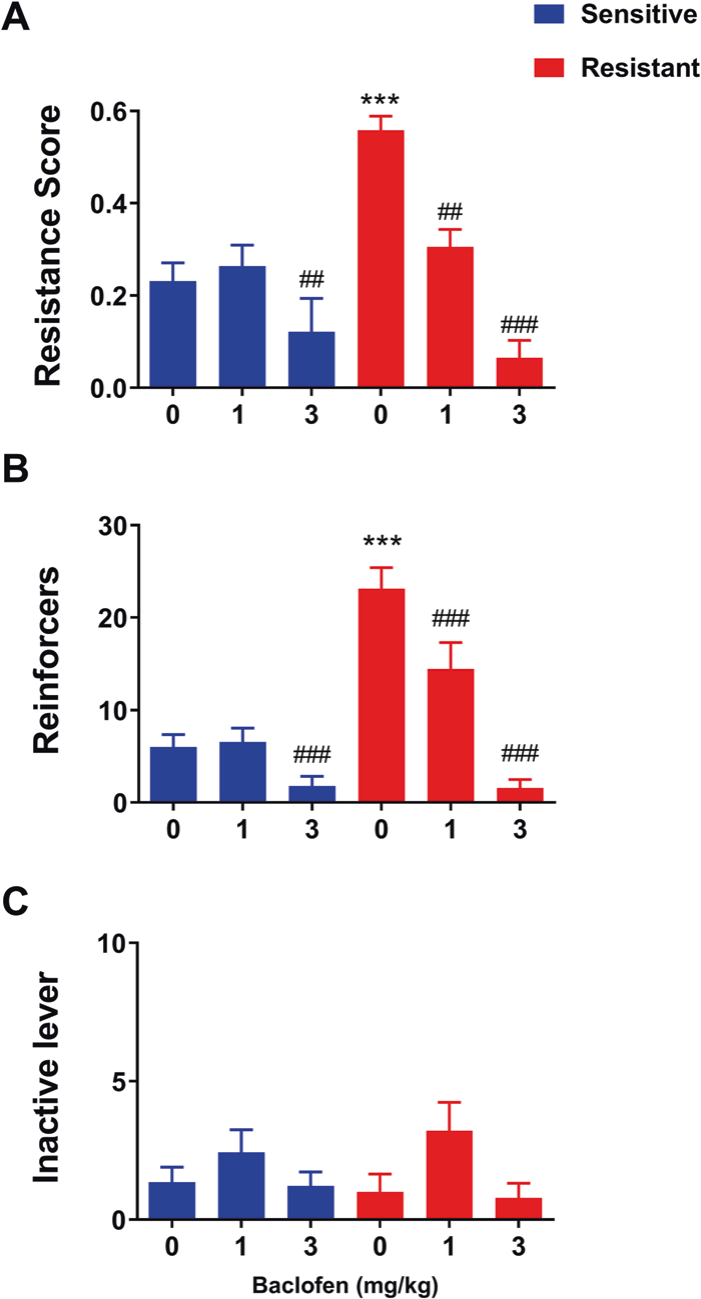
Systemic baclofen dose-dependently suppresses punished alcohol self-administration in punishment-resistant rats. **A** Mean resistance score ( ± SEM). **B** Mean number of alcohol reinforcers (±SEM) earned during the 30 min punishment session on FR2 schedule in punishment-resistant (*n* = 14) and -sensitive rats (*n* = 14) receiving baclofen (0, 1, 3 mg/kg). **C** Mean inactive lever presses (±SEM) during the 30 min test. ***p < 0.001 compared to shock-sensitive rats; ^##^*p* < 0.01, ^###^*p* < 0.001 compared to vehicle.

*Post-hoc* tests showed that punishment-resistant rats showed a higher resistance score and obtained a higher number of punished reinforcers (*p* < 0.001) compared to punishment-sensitive rats at the saline condition. Punishment-resistant rats showed lower resistance scores, and obtained a lower number of punished reinforcers, when treated with either baclofen dose compared to saline (Fig. 1A, B, *p* < 0.01, *p* < 0.001 for dose 1 and 3 mg/kg respectively). In contrast, punishment-sensitive rats showed lower resistance scores, and obtained a lower number of punished reinforcers only when treated with baclofen at the high, 3 mg/kg baclofen dose, compared to saline (Fig. 1A, B, *p* < 0.01, *p* < 0.001 respectively). No significant differences were detected on inactive lever responding (Fig. 1C).

### Intra-CeA baclofen administration decreases the activity of PKCδ+ CeA neurons in punishment-resistant rats

**Fig. 2:**
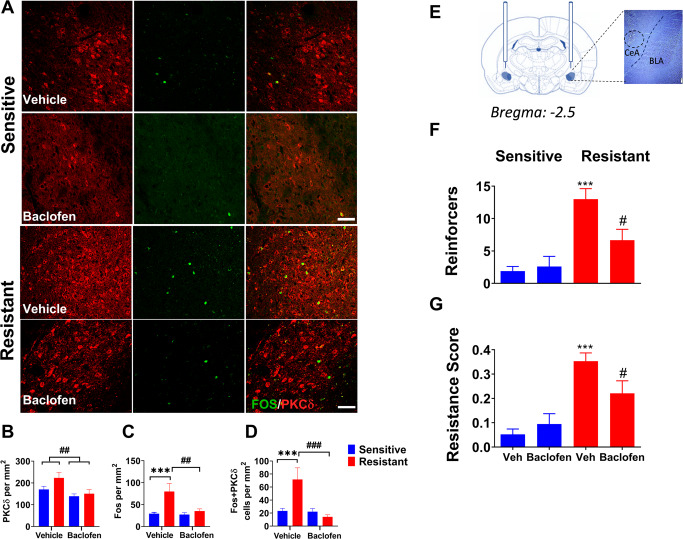
Intra-CeA baclofen administration attenuates resistance to punishment by decreasing activity of PKCδ+ CeA neurons. **A** Representative images of CeA photomicrographs showing PKCδ (red) and Fos (green) immunoreactivity co-labeling (scale bar, 50 μm), in punishment-sensitive and -resistant rats receiving intra CeA vehicle or baclofen microinjections (64 ng/side in 0.3 µl; *n* = 5/6 per group; injection site represented in the right upper panel). **B**–**D** Mean number of cells (±SEM) positive for PKCδ, Fos and double-labeled cells/mm^2^ in shock-sensitive and shock-resistant rats. ***p < 0.001 compared to shock-sensitive rats; ^##^*p* < 0.01, ^###^*p* < 0.001 compared to vehicle treated rats. **E** Baclofen injection site. **F** Mean number of alcohol reinforcers ( ± SEM) earned during the 30 min punishment session. **G** Mean resistance score in punishment-resistant (*n* = 18) and sensitive rats (*n* = 20) receiving vehicle and baclofen (64 ng/side; *n* = 9/10 per group). ***p < 0.001 compared to shock-sensitive rats; ^#^*p* < 0.05 compared to vehicle treated rats.

To test whether GABA_B_ receptor agonism suppresses the activity of PKCδ-expressing neurons that is associated with punishment-resistant alcohol self-administration, we microinjected baclofen into the CeA of punishment-resistant and punishment-sensitive rats. Activity of PKCδ−expressing neurons was indexed using the neuronal activity marker Fos. Punishment-resistant (*n* = 18) and -sensitive rats (*n* = 20) received baclofen (64 ng/side in 0.3 μl) or aCSF intra-CeA in a between subject design (9 and 10 per group, respectively), 15 min prior to the session. In agreement with our prior work, we detected increased Fos induction within PKCδ+ CeA neurons after punished alcohol self-administration in shock-resistant rats compared to the sensitive group (Fig. 2A–D, main effect of group -PKCδ: F_1,19_ = 3.37, *p* = 0.08; Fos: F_1,19_ = 13.3, *p* < 0.001; eta^2^ = 0.41; co-labelling: F_1,19_ = 4.5, *p* < 0.05; eta^2^ = 0.2). Intra-CeA baclofen microinjections decreased Fos expression in PKCδ+ neurons activated with punished alcohol self-administration (Fig. 2B–D, main effect of treatment- PKCδ: F_1,19_ = 8.77, *p* < 0.01; eta^2^ = 0.32; Fos: F_1,19_ = 7.7, *p* = 0.01; eta^2^ = 0.29; co-labelling: F_1,__19_ = 15.6, *p* < 0.001; eta^2^ = 0.45). The two groups responded differentially to treatment in Fos induction within PKCδ+ neurons (group x treatment interaction –PKCδ: F_1,19_ = 1.4, *p* = 0.25; Fos: F_1,19_ = 5.4, *p* < 0.05; eta^2^ = 0.22; co-labelling: F_1,19_ = 13.2, *p* < 0.01; eta^2^ = 0.41).

*Post-hoc* tests showed that punishment-resistant rats showed increased Fos induction and increased activity of PKCδ+ neurons compared to punishment-sensitive rats (*p* < 0.001). Baclofen treatment decreased expression of PKCδ in both punishment-resistant and -sensitive rats compared to aCSF (Fig. 2B, *p* = 0.01). Shock-resistant rats treated with baclofen showed decreased Fos induction and decreased expression of co-labelled cells compared to aCSF control group (Fig. 2C, D, *p* < 0.01; *p* < 0.001 respectively).

### Baclofen-induced decrease in PKCδ+ neuron activity is accompanied by suppression of punishment-resistant alcohol self-administration

In punishment-resistant rats, the intra CeA baclofen injections (Fig. 2E) also decreased the number of punished reinforcers obtained, and the resistance score. Factorial ANOVA showed a main effect of group (punishment -resistant vs. -sensitive) on the number of reinforcers obtained (F_1,34_ = 24.8, *p* < 0.001; eta^2^ = 0.42; Fig. 2F) and on resistance scores (F_1,34_ = 27, *p* < 0.001; eta^2^ = 0.42; Fig. 2G). There was a trend for a main effect of treatment on the number of reinforcers obtained, but not on the resistance score (F_1,34_ = 2.93, *p* = 0.1; F_1,34_ = 1, *p* = 0.3, respectively). Most importantly, there was a significant group x treatment interaction, both on the number of reinforcers obtained (F_1,34_ = 4.49, *p* < 0.05; eta^2^ = 0.1) and on the resistance scores (F_1,34_ = 4.1, *p* < 0.05; eta^2^ = 0.1). *Post hoc* tests showed that, following vehicle treatment, punishment-resistant rats obtained a higher number of punished reinforcers and showed higher resistance scores than shock-sensitive rats (*p* < 0.001). When treated with baclofen, punishment-resistant rats obtained a lower number of punished reinforcers and showed a lower resistance compared to the vehicle control condition (*p* < 0.05). There was no effect of group (F_1,34_ = 0.1, *p* = 0.78), treatment (F_1,34_ = 0.03, *p* = 0.86) or group x treatment interaction (F_1,34_ = 0.28, *p* = 0.59) on inactive lever response﻿s.

### GABA_B_ receptors are expressed by virtually all PKCδ neurons in CeA

**Fig. 3:**
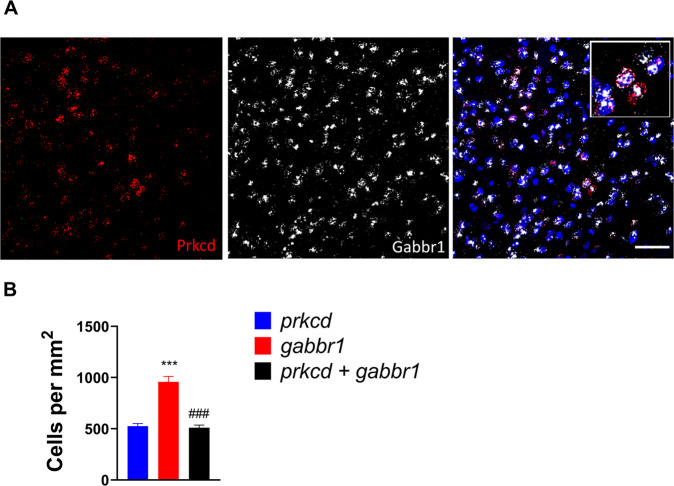
GABA_B_ receptors colocalize with PKCδ-expressing neurons in the CeA. **A** Expression of *Prkcd* and *gabbr1* in the CeA measured by RNAscope in situ hybridization. Red dots represent the expression of *Prkcd* and white dots represent the expression of *gabbr1* (scale bar, 50 um). **B** Mean number of cells positive per mm^2^ ( ± SEM) of *Prkcd, gabbr1* mRNA expression and their colocalization (*n* = 6 per group), ***p < 0.001 compared to *prkcd*, ^###^*p* < 0.001 compared to *gabbr1*.

To determine whether PKCδ neurons in CeA express GABA_B_ receptors, we analyzed the expression of transcripts encoding PKCδ (*prkcd*) and GABA_B_ (*gabbr1*), respectively, in the CeA of 6 naïve rats. *Prkcd* mRNA showed almost a complete overlap (~97.4 %) with *gabbr1* mRNA (Fig. 3A). One-way ANOVA showed a significant difference in the number of cells expressing the respective transcript (Figure 3B, F_2,15_ = 47.54, *p* < 0.0001; eta^2^ = 0.83). The number of *gabbr1* + was approximately twice as high as those that were *prkcd* + (*p* < 0.001), while the number of cells that co-expressed both transcripts and the number of *prkcd* + cells was virtually identical. These data show that almost all PKCδ + cells express the GABA_B_ receptor, but also that this receptor is expressed in other CeA neurons.

### Baclofen induces hyperpolarization of CeA neurons in a GABA_B_ -dependent manner

**Fig. 4:**
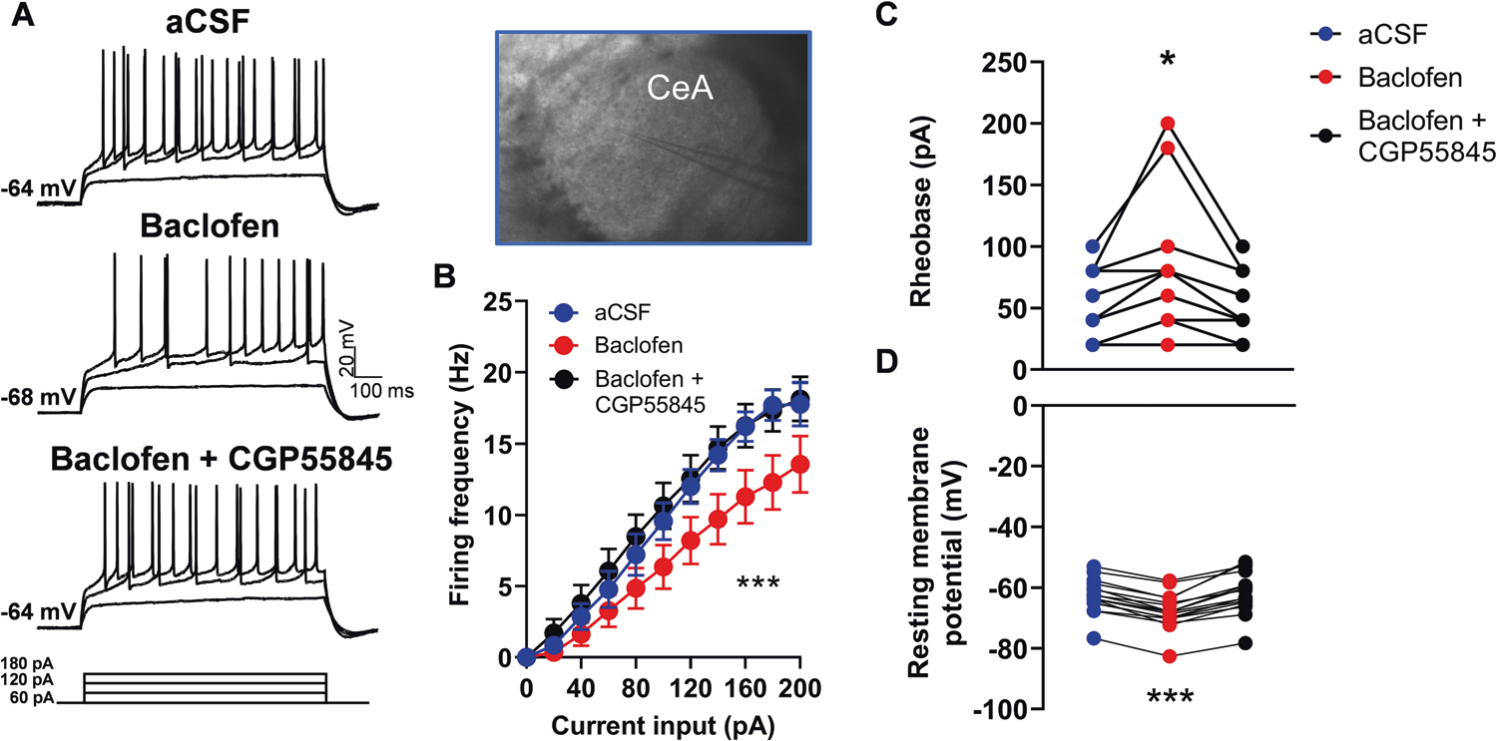
Baclofen induces hyperpolarization of CeA neurons in a GABA_B_-dependent manner. **A**, **B** Representative traces (left) and averaged action potentials discharge relative to current injections (right) obtained before (aCSF), and during subsequent baclofen (100 µM), and baclofen (100 µM) + CGP55845 (2 µM) perfusion in the CeA (*n* = 14 neurons from 6 rats). **C** Mean values of the rheobase ( ± SEM) in aCSF condition, during baclofen (100 µM), and baclofen (100 µM) + CGP55845 (2 µM) subsequent perfusions (*n* = 14 neurons from 6 rats). **D** Mean values of resting membrane potential ( ± SEM) in aCSF condition, during baclofen (100 µM), and baclofen (100 µM) + CGP55845 (2 µM) subsequent perfusions (*n* = 16 neurons from 6 rats). *p < 0.05, ***p < 0.001 baclofen vs control condition.

We next examined the effects of GABA_B_ receptor activation on the activity of CeA neurons. To this end, we performed patch-clamp electrophysiological recordings in CeA neurons from alcohol naïve rats. We found that baclofen (100 µM) decreased the action potential firing discharge in response to the injection of increasing amounts of depolarizing current (two-way repeated measures ANOVA: F_2,26_ = 21.89, *p* < 0.0001, *n* = 14 neurons from 6 rats). *Post hoc* test showed that baclofen perfusion significantly hyperpolarized CeA neurons (*p* < 0.001; Fig. 4A, B). Consistent with this finding, one-way repeated measures ANOVA showed a significant effect of baclofen on the rheobase (F_2,26_ = 10.59, *p* < 0.01, *n* = 14 neurons from 6 rats), and on the resting membrane potential (F_2,30_ = 63.86, *p* < 0.001, *n* = 16 neurons from 6 rats). *Post hoc* test showed that baclofen perfusion significantly increased the rheobase and decreased resting membrane potential (*p* < 0.05; *p* < 0.001, Fig. 4C, D). These effects were fully reversed by subsequent perfusion with the selective GABA_B_ receptor antagonist CGP55845 (2 μM; rheobase: *p* = 0.90; resting membrane potential: *p* = 0.20; Fig. 4C, D), demonstrating that baclofen reduced CeA neuronal activity in a GABA_B_ receptor-mediated manner. These findings show that baclofen produces a decrease in CeA neuronal activity.

### Effects of baclofen on control behaviors

**Fig. 5:**
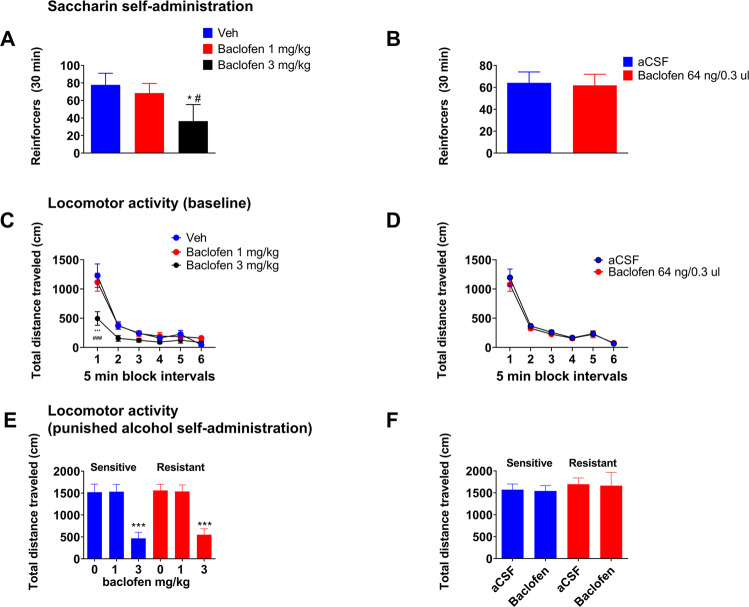
Effects of systemic or intra-CeA baclofen on saccharin self-administration and locomotor activity. **A** Mean number of reinforcers (±SEM) earned during 30 min of FR2 0.2% saccharin self-administration (*N* = 16) following either vehicle or baclofen (1, 3 mg/kg). *p < 0.05 baclofen 3 mg/kg vs control, ^#^*p* < 0.05 baclofen 3 mg/kg vs 1 mg/kg dose. **B**. Intra-CeA injection of baclofen (64 ng/side in 0.3 µl) did not affect saccharin self-administration (mean number of reinforcers ± SEM during 30 min). **C** Mean distance traveled ( ± SEM) (*N* = 27/9 per group). (***p < 0.001) baclofen 3 mg/kg compared to control and 1 mg/kg (^###^*p* < 0.001). **D** Intra-CeA baclofen injections with the same parameters did not affect locomotor activity (mean distance traveled ± SEM). **E** Mean distance traveled ( ± SEM) during 30 min of FR2 punished alcohol self-administration in shock-sensitive and shock-resistant rats (*n* = 14 / group) following either vehicle or baclofen (1, 3 mg/kg). ***p < 0.001, baclofen 3 mg/kg compared to vehicle treated rats. **F** Intra-CeA injection of baclofen (64 ng/side in 0.3 µl) did not affect locomotor activity (mean distance traveled ± SEM during 30 min punished alcohol self-administration) in shock-sensitive and shock-resistant rats (*n* = 9–10/group).

We assessed the behavioral specificity of baclofen effects on punishment-resistant alcohol self-administration using systemic as well as intra-CeA baclofen administration. The effect of systemic baclofen on saccharin self-administration was assessed in a new batch of rats (*N* = 16). After a stable baseline of responding for 0.2% saccharin was established, baclofen (0, 1, 3 mg/kg) was tested in a within subject design, with injections given 30 min prior to the saccharin sessions. Repeated measures ANOVA found a significant effect of treatment (F_2,30_ = 5.03, *p* = 0.01; eta^2^ = 0.25). *Post hoc* tests showed that baclofen at 3 mg/kg significantly reduced saccharin self-administration compared to both vehicle and 1 mg/kg (*p* < 0.05), while the 1 mg/kg dose did not differ from vehicle (Fig. 5A).

In a separate batch of rats (*N* = 20), we tested the effect of central baclofen microinjections on 0.2% saccharin self-administration. After a stable saccharin self-administration baseline was established, baclofen (64 ng/side in 0.3 µl) or aCSF, (*n* = 10/group) respectively, was administered into the CeA in a between subject design, 15 min prior to saccharin self-administration sessions. There was no difference in saccharin self-administration between baclofen and vehicle treated rats (Fig. 5B, Student’s t-test: t_18_ = 0.16, *p* = 0.87).

We then tested the effect of systemic baclofen on locomotor activity in a new batch of rats (*N* = 27). Baclofen was administered i.p. 30 min prior the test, using 0, 1, 3 mg/kg and a between subject design (*n* = 9/group). Two-way ANOVA showed a main effect of treatment (F_2,24_ = 5.13, *p* < 0.01; eta^2^ = 0.29), a main effect of time (F_5,120_ = 73.93, *p* < 0.0001; eta^2^ = 0.85) and a significant treatment x time interaction (F_10,120_ = 5.13, *p* < 0.001; eta^2^ = 0.29). *Post hoc* tests showed that baclofen at 3 mg/kg significantly reduced locomotor activity during the first 5 min bin compared to both vehicle and 1 mg/kg (*p* < 0.001). In contrast, locomotor activity in the 1 mg/kg dose was virtually indistinguishable from that of the vehicle group (Fig. 5C).

The effect of central baclofen microinjections on locomotor activity was assessed in the same set of rats previously tested on saccharin self-administration (*N* = 20) after one week of washout. Baclofen administration into the CeA with the parameters provided above did not affect locomotion. Specifically, on repeated measures of ANOVA, there was no main effect of treatment (F_1,18_ = 0.33, *p* = 0.57). There was a main effect of the time (F_5,90_ = 104.23, *p* < 0.001; eta^2^ = 0.85), but no treatment x time interaction (Fig. 5D, F_5,90_ = 0.35, *p* = 0.87).

The potential sedative effects of alcohol combined with baclofen was tested analyzing locomotor activity during punished alcohol self-administration sessions in vehicle and baclofen treated rats for both systemic and central injections. Two-way ANOVA with group as a between-subject factor (levels: punishment-resistant, punishment-sensitive) and treatment as a within-subject factor (levels: 0, 1, 3 mg/kg) showed a main effect of treatment (F_2,52_ = 40.27, *p* < 0.001; eta^2^ = 0.6), but no effect of group (F_1,26_ = 0.07, *p* = 0.78). Although there was no significant treatment x time interaction, *post hoc* tests showed that baclofen at 3 mg/kg significantly reduced locomotor activity in both groups compared to vehicle treatment (Fig. 5E, *p* < 0.001, while this was not the case following the 1 mg/kg dose). Locomotor activity after central injection of baclofen did not differ between groups (Fig. 5F, main effect of group: F_1,34_ = 0.43, *p* = 0.51; treatment: F_1,34_ = 0.03, *p* = 0.87 and interaction: F_1,34_ = 0; *p* = 0.9).

## Discussion

We show that, in the absence of physical dependence on alcohol, systemic administration of the prototypic GABA_B_ receptor agonist baclofen dose-dependently suppresses compulsive alcohol self-administration, a behavior operationally defined as self-administration that continues despite footshock punishment [7]. This effect was replicated by local CeA microinjections, and was likely mediated by effects on PKCδ+ neurons in CeA, whose activity was reduced by baclofen. Within CeA, virtually all PKCδ + neurons expressed GABA_B_ receptors, and patch-clamp recordings showed that baclofen hyperpolarizes CeA neurons in a GABA_B_ receptor-mediated manner. All our findings were obtained in male rats only, because we have not previously characterized individual variation in punishment-resistant alcohol self-administration in females; this characterization is presently ongoing.

The effects of baclofen on compulsive alcohol self-administration were behaviorally specific at the 1 mg/kg systemic dose, and also after CeA microinjections. In contrast, at the 3 mg/kg systemic baclofen dose, sedative effects began to emerge, as detected by decreased locomotor activity in drug naïve or alcohol self-administering rats and suppressed saccharin self-administration, similar to previous data on sucrose reinforcement [15]. It has previously been reported that baclofen administered at 3 mg/kg i.p. did not affect spontaneous motor activity in rats [20]. This apparent difference in sensitivity to sedative effects of baclofen may be related to the fact that we used outbred Wistar rats, while Colombo and colleagues evaluated baclofen in genetically selected alcohol preferring rats [20]. In humans, low sensitivity to sedative effects of alcohol itself correlate with genetic alcohol addiction risk [34, 35]. A differential sensitivity to sedative effects of baclofen between outbred rats and those genetically selected for alcohol preference may suggest that this generalizes to other sedatives, including GABA_B_ acting drugs.

Our findings of suppressed compulsive alcohol self-administration following systemic baclofen administration are consistent with those recently reported by others, using another model of alcohol taking despite negative consequences, or despite the availability of an alternative sweet reward. In that model, where compulsivity is operationally defined as drinking that is resistant to quinine adulteration, or to alternative saccharin reward, baclofen at systemic doses similar to ours selectively decreased compulsive alcohol drinking in rats resistant to adulteration or alternative reward [8]. This is in line with our previous findings that footshock punishment -resistant alcohol self-administration and alcohol drinking resistant to quinine adulteration and to alternative saccharin reward are strongly correlated [7, 10], suggesting that they share an underlying neurobiological mechanism. The consistency of findings with baclofen across these models further strengthens this notion, and provides support for the ability of GABA_B_ activation to suppress punishment resistance towards alcohol. It was recently reported that punishment resistance towards alcohol drinking is predicted by the emergence of habitual alcohol seeking behavior [36]. Because our procedure does not allow seeking and taking to be isolated from each other, our data do not address the question whether baclofen targets seeking or taking behavior, or both.

Our findings point to the CeA as the site of action for baclofen to suppress compulsive alcohol self-administration, and suggest that inhibition of activity in CeA neurons mediate this effect. Specifically, in a replication of our previous study [7], we found an association between activity of PKCδ+ neurons within the CeA, as indexed by Fos-expression, and compulsive alcohol self-administration. Intra-CeA microinjections of baclofen markedly decreased this PKCδ+ neuron activity, and this was accompanied by suppression of compulsive alcohol self-administration.

GABA_B_ agonists such as baclofen can influence neuronal activity by inhibiting activity-dependent neurotransmitter release from presynaptic axon terminals, or by hyperpolarizing postsynaptic neurons through activation of inwardly rectifying ion channels [37]. Previous findings have shown that baclofen acts presynaptically in the CeA to reduce neurotransmitter release at inhibitory terminals [38]. Here, we examined whether direct, postsynaptic GABA_B_ effects on PKCδ+ neuron activity might be involved in the neural and behavioral effects of baclofen. As a prerequisite for this, PKCδ+ neurons would have to express GABA_B_ receptors. We found that this is indeed the case, as virtually all, or ~97.4% of PKCδ + CeA neurons expressed GABA_B_ receptors in our co-expression analysis. Of note, GABA_B_ receptors were also expressed by an equally sized population of CeA cells that were not PKCδ+. We did not examine the phenotypic identity of these cells, but its size suggests that it is likely to include somatostatin-expressing (SOM +) GABA neurons, as PKCδ+ and SOM + cells together account for appr. 80% of CeA neurons [39]. Additionally, CRF + neurons might indirectly affect PKCδ activity, as it has been shown that knockdown of the vesicular GABA transporter in CeA CRF neurons increases Fos expression in PKCδ+ cells [40].

We then found that baclofen perfusion potently and concentration-dependently hyperpolarized CeA neurons, resulting in a reduction of their firing rates. These effects were GABA_B_ receptor mediated, as they were abolished by perfusion with the selective GABA_B_ antagonist CGP55845. These effects of baclofen are consistent with a direct activation of postsynaptic GABA_B_ receptors in CeA, and point to a novel mechanism for GABA_B_ agonists in this structure. A limitation of our study is that we did not establish the phenotypic identity of the CeA cells that were inhibited. Since we found that GABA_B_ receptors are expressed by a majority of CeA neurons, we cannot exclude that effects of baclofen to suppress compulsive alcohol self-administration might be indirect, and involve presynaptic suppression of GABA release from upstream neurons.

However, we believe data collectively suggest this to be unlikely. We previously showed that DREADD-mediated inhibition of PKCδ+ neuronal ensembles is sufficient to suppress compulsive self-administration [7]. In the present study, a similar inhibition of PKCδ+ neuron activity was obtained with baclofen, and had the same behavioral consequences. Meanwhile, the slice experiments demonstrated an ability of baclofen to inhibit CeA neurons through a direct, postsynaptic mechanism. Collectively, these findings are consistent with the interpretation that baclofen may be able to inhibit CeA neurons from multiple populations, but will preferentially inhibit ensembles of PKCδ+ neurons that, when activated, promote compulsive alcohol self-administration, thus suppressing this behavior.

Our findings are broadly consistent with, and expand on an extensive literature of human and animal studies suggesting a key role for CeA in alcohol dependence [10, 11, 41–44]. It was shown early on that CeA lesions reduce alcohol drinking in rats [42]. More recent studies have shown that activity of neuronal ensembles within CeA causally contributes to alcohol withdrawal-induced behaviors [43] and compulsive alcohol self-administration [7]. A recent single-nucleus RNA sequencing study also identified PKCδ + CeA neurons as the subpopulation in which the most extensive gene expression changes occurred during alcohol withdrawal [45]. Moreover, a subpopulation of PKCδ + cells in the CeA, characterized by co-expression of PKCδ and the neuropeptide cocaine and amphetamine regulated transcript (CART), was recently shown to mediate stress-induced alcohol seeking [46].

In summary, using baclofen as a tool compound, we provide preclinical data in support of the notion that GABA_B_ receptor activation has therapeutic potential to suppress compulsive alcohol use. Following a seminal positive study [47], multiple clinical trials have been carried out to evaluate the efficacy of baclofen in clinical alcohol use disorders. Results have been somewhat variable, but a meta-analysis has found overall support for efficacy of baclofen [21], and this conclusion has since been further strengthened [48].

Despite these promising findings, baclofen has essential limitations as a therapeutic. As can be expected with an orthosteric agonist, its chronic dosing frequently results in tolerance, dose escalation and adverse events. In the present study, acute baclofen administration allowed us to establish a mechanistic role of GABA_B_ receptors in individual susceptibility to compulsive alcohol use. To overcome the limitations of baclofen as a therapeutic for chronic, clinical use, positive allosteric modulators of the GABA_B_ receptor offer an approach with the potential to achieve similar therapeutic effects, while avoiding baclofen’s limitations [49].
